# Topiramate causing type II renal tubular acidosis: A case and review of the mechanism

**DOI:** 10.1002/ccr3.2733

**Published:** 2020-02-18

**Authors:** Michael Chahin, Monique Oye, Sonal Jadeja, Kimberly Sanders, Pramod Reddy

**Affiliations:** ^1^ Department of Internal Medicine University of Florida College of Medicine ‐ Jacksonville Jacksonville FL USA

**Keywords:** carbonic anhydrase inhibitor, proximal renal tubular acidosis, topiramate

## Abstract

Topiramate has a wide array of pharmacologic effects, including proximal renal tubular acidosis (RTA). Clinicians must be wary of the possibility for development of somnolence due to compensatory hyperventilation and cardiac dysrhythmias.

## Graphical abstract

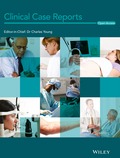

## 1 INTRODUCTION

Topiramate is an under‐recognized cause of proximal renal tubular acidosis. Our patient was started on topiramate and developed a normal anion gap metabolic acidosis that was discovered after an overdose attempt. The diagnosis was confirmed by bicarbonate challenge. Patients taking topiramate may become somnolent, and respiratory failure has been reported.

Type II, or proximal, renal tubular acidosis (RTA) occurs due to loss of bicarbonate (HCO3‐) reabsorption in the proximal tubule.^1^ Type II RTA is frequently attributed to etiologies such as hyperparathyroidism and multiple myeloma.^2^ Topiramate is an anticonvulsant with a wide range of pharmacological activity, including reduction in voltage‐sensitive sodium channel activity, increased *γ*‐aminobutyric acid (GABA)_A_ receptor activity, and inhibition of carbonic anhydrase.^3^ We present a patient who developed type II RTA after being initiated on this medication.

## 2 CASE REPORT

An 18‐year‐old female with past medical history of asthma and bipolar depression presented to the emergency department after an overdose attempt with topiramate and atomoxetine. In addition to suicidal ideation, she complained of headache and fatigue. Her physical examination was significant for somnolence, lateral nystagmus, and increased respirations. She was not in respiratory distress. Her QTc interval was 508 ms Acetaminophen, aspirin, ethanol, and ketone levels were negative. Her serum osmolality was normal, and her topiramate level was 70.5 ug/mL. She had reportedly been taking lamotrigine, but testing was negative. Serum bicarbonate was 16 mmol/L, with a normal anion gap, and potassium was 3.3 mmol/L. Urine pH was 5.5.

Renal function testing revealed elevated creatinine (1.02‐1.23 mg/dL) from a baseline of 0.71 mg/dL and a normal anion gap metabolic acidosis with respiratory compensation. Over the past year, serum bicarbonate ranged from 15 to 18 mmol/L and potassium was 3.3‐3.8 mmol/L. She had been started on topiramate 200 mg daily for weight loss just prior to this period. During an admission two months before this one, also after a topiramate overdose, she was treated with sodium bicarbonate tablets for normal anion gap metabolic acidosis with no improvement in her serum bicarbonate and resulting in alkalotic urine pH. Urine pH at that time was 7, whereas it was 5.5 before. She reported having continued to take the medication, this was confirmed by the pharmacy.

Over several hours, her symptoms resolved. Repeat electrocardiogram revealed QTc interval of 460 ms She was advised to stop taking topiramate and trialed on bicarbonate supplementation for several days. She did not have recurrence of somnolence, or other symptoms, during the remainder of the admission. Repeat laboratories two months later revealed resolution of the metabolic acidosis, after cessation of topiramate.

## 3 DISCUSSION

Type II (Proximal) renal tubular acidosis is caused by decreased HCO3‐ reabsorption in the proximal tubule, which is the primary site of its reabsorption in the kidney.^4^ Etiologies can be divided between causes of selective and diffuse type II RTA, which depend on the defects present in the proximal tubule. For example, Fanconi syndrome causes the diffuse type, as evidenced by the presence of glycosuria despite normal serum glucose and amino aciduria. Acetazolamide is a classic example of selective impairment of HCO3‐ reabsorption since there this is the only defect and there is no loss of glucose or amino acids.^5^ Topiramate also causes selective type II RTA by inhibiting carbonic anhydrase II, the predominant renal isoenzyme of carbonic anhydrase.^4^, ^6^

In type II RTA, the reabsorption of HCO3‐ in the distal tubule is unaffected. Ordinarily, the threshold for HCO3‐ loss in adults in the proximal tubule is 26 mmol/L. However, in these patients there is a compensatory excretion of ammonium (NH4+) to match the decreased serum HCO3‐.^1^ Thus, the threshold for HCO3‐ release is approximately 15 mmol/L. Type II RTA can be divided into active and maintenance phases, which is not frequently mentioned in the literature. The active phase occurs during this equalization, which leads to a hyper‐chloremic metabolic acidosis. HCO3‐ loss leads to volume contraction causing the development of a hyper‐aldosterone state and chloride reabsorption.^7^

The maintenance phase occurs once the lower HCO3‐ threshold is established.^7^ The diagnosis can be made with bicarbonate loading, such as seen in our patient.^8^ Her urine pH became alkalotic and the serum bicarbonate was relatively unchanged, as a result of this compensation. Between the two admissions for topiramate overdose, she had two confirmatory bicarbonate challenge tests. Had it been measured her urine bicarbonate would have been elevated.

Indications for topiramate include the treatment of epilepsy and migraine prophylaxis.^9^ However, it has been used off‐label, such as in our patient, for weight loss.^10^ Type II RTA due to topiramate has been demonstrated in the literature but is still likely an underreported issue.

Clinicians must be wary of the effects of this metabolic acidosis, which can include hyperventilation, fatigue, cardiac dysrhythmias, and poor bone health. In addition to proximal RTA, topiramate causes hypocitraturia and thus increased risk for nephrolithiasis.^11^ Zonisamide and acetazolamide have also been identified as causing decreased urine citrate, and this is a unique characteristic from other causes of proximal RTA.^12^

A case series demonstrated the symptoms of topiramate overdose include somnolence, agitation, vertigo, and mydriasis, whereas some patients were asymptomatic. The asymptomatic patient ingested a relatively low dose, 10.7 mg/kg, compared to the others in the series, and was treated with gastric lavage within one hour.^13^ There is another case of a woman who was on topiramate for several months who presented with dyspnea and ultimately required intubation for respiratory fatigue. She was found to have a normal anion gap metabolic acidosis, and her condition improved after cessation of the medication.^14^

Our patient had also taken atomoxetine in excess, which likely contributed to her drowsiness and lateral nystagmus. However, the acidosis exhibited by tricyclic antidepressant consumption has been attributed to lactic acidosis and respiratory depression.^15^

## 4 CONCLUSION

Topiramate is a commonly prescribed medication that has demonstrated efficacy in epilepsy and migraines. As further uses are elucidated, it is crucial that prescribers are aware of the development of metabolic acidosis due to carbonic anhydrase impairment. Routine metabolic and urine testing is essential when starting patients on this medication as it may cause severe adverse effects.

## CONFLICT OF INTEREST

None declared.

## AUTHORS' CONTRIBUTIONS

MC: described and formatted the case. MC and MO: collaborated on the literature review. KS and SJ: edited the manuscript. PR: served as the advisor and was the clinician who treated the patient.
